# Traditional Chinese Medicine Body Constitutions as Predictors for Depression: A Systematic Review and Meta-Analysis

**DOI:** 10.3390/bs12110423

**Published:** 2022-10-30

**Authors:** Sin Yee Yap, Foong Leng Ng, Menaga Subramaniam, Yang Mooi Lim, Chai Nien Foo

**Affiliations:** 1 Centre for Cancer Research, M. Kandiah Faculty of Medicine and Health Sciences, Universiti Tunku Abdul Rahman, PT21144, Jalan Sungai Long, Bandar Sungai Long, Kajang 43000, Selangor, Malaysia; 2 Department of Traditional Chinese Medicine, M. Kandiah Faculty of Medicine and Health Sciences, Universiti Tunku Abdul Rahman, PT21144, Jalan Sungai Long, Bandar Sungai Long, Kajang 43000, Selangor, Malaysia; 3 Department of Pre-Clinical Science, M. Kandiah Faculty of Medicine and Health Sciences, Universiti Tunku Abdul Rahman, Lot PT21144, Jalan Sungai Long, Bandar Sungai Long, Kajang 43000, Selangor, Malaysia; 4 Department of Population Medicine, M. Kandiah Faculty of Medicine and Health Sciences, Universiti Tunku Abdul Rahman, PT21144, Jalan Sungai Long, Bandar Sungai Long, Kajang 43000, Selangor, Malaysia

**Keywords:** traditional Chinese medicine, body constitution, depression, predictor, systematic review, meta-analysis

## Abstract

Traditional Chinese medicine body constitution (TCMBC) reflects a person’s vulnerability to diseases. Thus, identifying body constitutions prone to depression can help prevent and treat depression. The review aimed to assess and summarize the existing evidence that explores the relationship between TCMBC and depression. Psychology and Behavioral Sciences Collection, MEDLINE, PubMed, CNKI, Wanfang, SinoMed, Embase, VIP, CINAHL, and CMJ were searched from inception to April 2021. Observational studies assessing the association between TCMBC and depression were selected. The quality of the included studies were assessed using the Newcastle–Ottawa Scale (NOS). Eighteen studies were included in the systematic review and thirteen in the meta-analysis. The pooled odd ratios of developing depression for Qi-stagnation, Qi-deficiency, Yang-deficiency, Yin-deficiency, and Balanced constitutions were 3.12 (95% CI, 1.80–5.40; I^2^ = 94%), 2.15 (95% CI, 1.54–3.01; I^2^ = 89%), 1.89 (95% CI, 0.71–5.03; I^2^ = 81%), 1.41 (95% CI, 0.91–2.20; I^2^ = 57%), and 0.60 (95% CI, 0.40–0.90; I^2^ = 94%), respectively. The findings suggest that the evaluation of a person’s TCMBC could be useful the in prevention and treatment of depression. However, more case-control and cohort studies are required to further confirm the association between TCMBC and depression.

## 1. Introduction

Depression is the cancer of the 21st century. It is one of the leading causes of the overall global burden of disease [1]. As of 2017, about 264 million people suffered from depression globally, with a higher prevalence in women (4.1%) than men (2.7%) [1]. Depression often develops at a young age and is constantly recurring [2]. Depression is not merely excessive sadness, but rather, a combination of factors related to negative thoughts, other symptoms and the bodily impact that lead to significant impairments in how an individual functions in daily life. Depressed individuals are shown to be vulnerable to heart diseases [3], diabetes [4], stroke [5] and infectious diseases [6]. Depression is a significant cause of mortality [7] and an important risk factor for suicide. According to the World Health Organization (WHO), nearly 800,000 people die due to suicide each year, which means that every 40 s, a person kills him/herself. Globally, suicide is the second leading cause of death in children, adolescents and young adults [8].

There are variations in the types of depression and their severities. The most common type of depression is major depressive disorder (MDD), also known as clinical depression. It is characterized by depressed mood and loss of interest or pleasure [9]. According to the fifth edition of the Diagnostic and Statistical Manual of Mental Disorders (DSM-5), at least one of these two symptoms must be present along with another five or more symptoms for at least two weeks for a person to be diagnosed with MDD. Several other symptoms include sleeping problems, changes in appetite, constant fatigue, difficulty concentrating, agitation or slowed movement, feeling guilty or worthlessness, unexplainable pains and suicidal thoughts [9]. Dysthymia, also known as a persistent depressive disorder, is an ongoing and chronic form of depression. Its symptoms are often less severe than MDD but longer lasting. The essential feature of this disorder is the presence of a sad mood on most days for at least two years [9]. Besides, some people may experience seasonal affective disorder (SAD) during fall or winter due to reduced daylight [9]. SAD usually wears off during Spring and Summer. The main symptoms include social withdrawal, oversleeping, low energy and weight gain [9]. Another subtype of depression is bipolar disorder, which also called manic depression. People who suffer from bipolar disorder can have extreme mood swings from emotional highs to lows [9]. During the low phases, they will experience symptoms of MDD.

Depression is often caused by a combination of various factors, rather than just one cause. There is a range of contributing factors that can lead to depression. The genes and traits that one inherits from their parents make them prone to depression [10]. Lack of social support, troubled relationships or loss of loved ones can also induce suicidal thoughts and feelings of worthlessness, increasing depression risk [10]. Other risk factors for depression include stressful life events, childhood trauma, substance use, poor nutrition and lack of exercise [10]. This is consistent with past reviews and meta-analyses that found social support [11,12,13], substance use [14], diet [15], physical activity [16] and exposure to early life stress, such as childhood trauma and loss of loved ones [17], were associated with depression risk. In addition, depression is also a common complication of other chronic illnesses. For instance, a recent Danish study showed that people who suffered from heart diseases and stroke were more likely to have subsequent depression [18].

Currently, the screening and diagnosis of depression is mainly based on symptoms. Psychiatrists diagnose depression according to patients’ descriptions of symptoms, questionnaires and clinical behaviour observations, and subsequently categorize the patients according to the DSM-5 [9] and the eleventh revision of International Statistical Classification of Diseases and Related Health Problems (ICD-11) [19]. There is no laboratory test to identify depression due to its heterogeneous nature. The complex interaction of genetic, biological, psychological and environmental factors that contribute to depression affects the accuracy of diagnosis, our understanding towards its pathophysiology, and our ability to develop effective treatments.

Depression is treatable; however, many depressed individuals fail to receive adequate treatment, especially those in low- and middle-income countries [20]. Barriers to effective care include inaccurate diagnosis, lack of facilities and trained personnel, social discrimination and high treatment costs [21]. Treatments of depression usually include medications and psychotherapies. There are several types of antidepressants available, such as selective serotonin reuptake inhibitors (SSRIs), tricyclic antidepressants (TCAs) and monoamine oxidase inhibitors (MAOIs) [9,22]. However, these drugs may induce a range of side effects, such as dry mouth, vision problems, dizziness, irritability, bleeding abnormalities, seizure and constipation [9,22,23]. Psychotherapies are also known as talk therapies. Examples of psychotherapies are cognitive-behavioral therapy (CBT), interpersonal therapy (IPT) and problem-solving therapy [9,24]. Past meta-analyses demonstrated that pharmacotherapy [25,26] and psychotherapy [25,27,28] were associated with reduced risk of relapse and recurrence in depression.

Traditional Chinese medicine (TCM) is one of the oldest medical systems globally. An important aspect of TCM is the prevention of diseases by maintaining or restoring the harmony and equilibrium of Yin-Yang within the human body [29,30]. Illness often occurs due to the imbalance of Yin-Yang. According to TCM theories, the five fundamental substances (essence, Qi, blood, body fluids and spirit) and the five viscera (liver, spleen, lung, heart and kidney) are closely related to each other and the formation of body constitution [31]. Biased body constitutions result from the impaired viscera function and dysregulation of fundamental substances [31]. The concept of Traditional Chinese medicine body constitution (TCMBC) reflects a person’s unique physical, physiological and psychological functions [32]. It is determined by hereditary and acquired factors in the process of human life [32]. Pathologically, TCMBC also influences a person’s susceptibility to certain pathogenic factors and diseases, as well as their reaction to treatment [32]. It is the foundation for TCM practitioners to diagnose, treat and prevent diseases [33]. TCMBC is alterable due to its relative stability and dynamic variability [34]. A biased constitution can be modified towards a neutral type through acquired factors such as exercise and diet. An appropriate amount of physical activity can produce strong muscles and bones, promote the blood circulation and Qi dynamic and enhance visceral function [32]. This can then prevent the formation of biased constitutions, such as Blood-stasis (BSC) and Qi-stagnation (QSC). On the contrary, lack of exercise will result in flabby muscles, restricted flow of Qi and blood and impaired spleen and stomach function, which can contribute to the formation of a Phlegm-dampness constitution (PDC) [32]. Furthermore, a healthy diet and sufficient nutrients can produce a strong physique and good constitution, while an unbalanced diet and malnutrition could lead to a weaker constitution [32]. Very often, biased and unbalanced constitutions are detected among depressed populations [35,36,37]. For example, Chen et al. found that women with Yang- and Yin-deficient constitutions had a higher risk of depression [35], while Xiong et al. found that college students with Qi-stagnation and Qi-deficiency constitutions were more prone to depression [38].

The China Association for Traditional Chinese Medicine classified TCMBC into nine types, namely the Balanced constitution (BC), Qi-stagnation constitution (QSC), Blood stasis constitution (BSC), Qi-deficiency constitution (QDC), Yin-deficiency constitution (YIDC), Yang-deficiency constitution (YADC), Phlegm-dampness (PDC), Damp-heat constitution (DHC) and Inherited special constitution (ISC) [34]. Among them, BC is a neutral type, while the rest are biased and unbalanced types. BC is a harmonious constitution, with a balance of Yin-Yang [32,39]. People with this constitution display common features, such as a normal body shape, strong physique, optimistic personality, good adaptability, energetic nature and strong immune system [31,32,40]. People with BC seldom get sick, and if they do, they recover from sickness easily [32]. Generally, people with QSC often cope poorly with stressful situations. People with this constitution exhibit a thin physique, mood swings, suspiciousness, overthinking and excessive worrying [31,32,40]. People with BSC usually have dull skin and dark lips, get bruises easily, are forgetful and are averse to cold environments and weather [31,32,40]. Additionally, they are prone to body pain and bleeding [31,32]. Next, people with QDC are easily exhausted due to weak immunity [31,32,40]. They are prone to panting and colds, and are easily affected by sudden climate changes. These people require a longer time to recover from sickness [31,32]. People with YIDC have warm palms and soles, are impatient and exhibit an extroverted nature [40]. These people are always thirsty, prefer cold drinks and dislike hot and dry weather [31,32]. In contrast, people with YADC are usually introverted, quiet, shy and have cold limbs [40]. They prefer hot meals and summer over winter [31,32]. The main characteristics of PDC include excessive phlegm production, overweightedness, chest tightness and a mild-mannered and patient nature [31,32,40]. These people like high sugar and high fat food and dislike damp environments [31,32]. People with DHC usually have oily skin, are prone to acne outbreaks, have a bitter taste in their mouths, and experience difficult and sticky bowel movements [31,32,40]. They are irritable and averse to hot and humid climates [32]. Lastly, people with ISC often have an inherent sensitivity to certain allergens, such as pollen, odors, food and medicines [40]. They tend to have conditions like asthma and are sensitive to environmental changes [31,32].

Conventionally, TCM practitioners describe the etiologies and symptoms of depression caused by extreme emotional changes using “yu” or “yuzheng”, which means blockage, stagnation, not flowing, clogging, or obstruction [41]. In TCM, the deficiency of Qi (vital energy) is believed to be the main cause of depression [42,43]. Qi deficiency could be due to physiological dysfunctions in the human body, which include inflammation, abnormal blood circulation, formation of dampness or phlegm [43]. Hence, strengthening the Qi and fixing imbalances of the physiological systems are the principles for healing depression [44]. In TCM, the liver is in charge of dispersion and dredging to regulate digestion, absorption and emotions, as well as the circulation of Qi, blood and body fluids [31,45]. Normally, the liver-Qi is the first to be affected directly during an emotional change, followed by disharmony of the Qi among the five viscera, which can then lead to the dysregulation of the Qi and blood [43]. A dysfunction of liver dispersion and dredging can also lead to the repression of spleen function, followed by the dysregulation of heart-Qi, then leading to the “shen” (spirit) becoming restless, which can result in an unstable and depressed mood [43]. This is because our spirit resides in the heart, and heart-Qi is in charge of pumping blood and the regulation of blood flow within the human body [31]. Past studies have confirmed this theory, where the abnormal dispersion of liver-Qi causes depression [46,47,48,49,50,51].

Currently, application and research on TCMBCs are mainly performed in Asian countries, such as China [35,36,37,38], Japan [52,53], Hong Kong [54,55], the Philippines [56] and Malaysia [57]. Identification of TCMBCs that are vulnerable to depression can allow us to modify them towards harmony and balance. TCMBC has clinical significance in preventing depression as it can be applied to indicate a person’s overall health conditions and help prevent depression in the early phase. With the extensive application of TCMBC in the past decade, a number of studies have revealed that depression is correlated to TCMBC [58,59,60,61,62]. However, the findings of the associations are inconsistent and lack a systematic review to clarify the strength of these associations. Only a narrative review reporting the potential role of TCMBC in the development of depression was published [63]. Hence, there is a need for a comprehensive review to evaluate the association between TCMBC and depression. To date, and to our knowledge, this is the first systematic review and meta-analysis investigating the association between TCMBC and depression. This systematic review and meta-analysis aims to assess and to summarize the existing empirical data that explored the relationship between TCMBC and depression. The key objectives are as follows: (1) to report whether TCMBC is associated with depression; and (2) to assess whether TCMBC predicts depression. The findings of this review will provide knowledge and references for developing measures to manage depression.

## 2. Materials and Methods

The conduct and reporting of this systematic review and meta-analysis were strictly based on the Preferred Reporting Items for Systematic reviews, and Meta-Analyses (PRISMA) [64] and Meta-analysis of Observational Studies in Epidemiology (MOOSE) [65] guidelines, following an a priori protocol. The study protocol was registered and published at the International Prospective Register of Systematic Reviews (PROSPERO) with a registration number of CRD42021267651, and is under review for publication.

### 2.1. Data Sources and Search Strategy

Comprehensive literature searches were conducted in the following databases: Psychology and Behavioral Sciences Collection, MEDLINE, PubMed, Chinese National Knowledge Infrastructure (CNKI), Wanfang, SinoMed, Embase, Chinese Scientific Journal Database (VIP), Cumulated Indexed to Nursing and Allied Health Literature (CINAHL) and Chinese Medical Journal Database (CMJ). No restriction was set on the publication date. The database searches were limited to journal articles written in the English and Chinese languages only. The database searches were conducted from December 2020 to April 2021. The search terms used are presented in Table 1. Additionally, the references of the included studies were manually searched to identify other relevant studies.

### 2.2. Eligibility Criteria

The main inclusion criteria were related to: (1) study type: observational studies including cohort, case-control and cross-sectional studies that investigate the association between TCMBC and depression; (2) participant: all subjects and populations were considered; if there was a control group, the subjects should be from the general population and without depression; (3) outcome: the correlation between TCMBC and depression were reported; (4) measurement of TCMBC and depression: the identification of TCMBC and depression through validated instruments. Only articles published in English and Chinese were included.

The exclusion criteria were as follows: (1) was not a journal article (e.g., conference abstract, dissertations and reports); (2) was not primary research (e.g., systematic review and meta-analysis); (3) lacked sufficient information to determine eligibility; (4) involved non-human subjects; (5) did not explicitly focus on the association between TCMBC and depression.

### 2.3. Study Selection

For English databases (Psychology and Behavioral Sciences Collection, MEDLINE, PubMed, and Embase), two reviewers (SYY and MS) independently conducted the searches and screened the titles and abstracts of all retrieved articles, followed by the full text screening of potentially eligible studies. For Chinese databases (CNKI, Wanfang, SinoMed, VIP, CINAHL, and CMJ), two reviewers (SYY and FLN) independently conducted the searches and screened the titles and abstracts of all retrieved articles, followed by the full text screening of potentially eligible studies. The full texts were reviewed according to predefined inclusion criteria. Disagreements at both screening levels (title/abstract and full text) were resolved through discussion and consultation with other authors (YML and CNF).

### 2.4. Data Extraction

Three reviewers (SYY, FLN and MS) independently extracted the data from the included studies using a standardized data extraction spreadsheet. Disagreements were resolved through discussion with other authors (YML and CNF). The following data were extracted: first author, year of publication, study design, study subjects, sampling method, study location, sample size, age, gender, ethnicity, depression measurement, TCMBC measurement, type of constitutions studied, main results (e.g., *p* value, odd ratio (OR) and 95% confidence interval (CI)). The primary outcome was the association between TCMBC and depression.

### 2.5. Missing Data

When encountering missing data, the corresponding authors of the potentially eligible studies were contacted by E-mail to retrieve further data or clarifications. The studies were excluded and the data synthesis was conducted using available data when the authors did not respond or failed to provide the relevant data requested within a month.

### 2.6. Assessment of Risk of Bias and Certainty of Evidence

Three reviewers (SYY, FLN and MS) independently performed risk of bias assessment using the Newcastle–Ottawa Scale (NOS) [66,67]. The NOS evaluates the quality of the included studies regarding three main aspects: (1) selection; (2) comparability; (3) exposure. The maximum scores for case-control and cohort studies are 9 and for cross-sectional studies are 10. Regarding the quality of the included case-control and cohort studies, they were considered as poor if the score was 0 to 5 and good if the score was 6 to 9. For the quality of cross-sectional studies, they were rated as poor if the score was 0 to 4, medium if the score was 5 to 6, good if the score was 7 to 8 and very good if the score was 9 to 10. Disagreements in the quality assessment were adjudicated by discussion with other authors (YML and CNF).

The quality of evidence and the strength of recommendation of this review were evaluated using the Grading of Recommendations Assessment, Development and Evaluation (GRADE) guidelines [68]. Five criteria were considered when decreasing the level of certainty, including risk of bias, imprecision, inconsistency, indirectness and publication bias. Whereas, three additional criteria, which included large magnitude of effect, dose-response gradient, and when residual confounders would decrease the magnitude of effect (when an effect is observed), were considered when upgrading the level of certainty. The overall quality can be rated as very low, low, moderate and high. The lowest quality of evidence for any of the outcomes determine the overall quality of evidence.

### 2.7. Data Synthesis and Analysis

Data from the eligible studies were summarized descriptively in tabular format and narrative text. The characteristics of the studies were reported and grouped in the table based on population types (diseased and general populations). Cochrane Software Review Manager (RevMan), version 5.3 (The Cochrane Collaboration, 2020) was used to perform statistical analysis if a meta-analysis was allowed. Meta-analyses were performed if there was sufficient number of studies (*n* ≥ 2), adequate quality of studies (moderate and good quality) and similarity in the study design. Inverse variance analysis was used in the meta-analyses. The extracted ORs and 95% CIs were converted to log ORs and standard errors (SE) using the RevMan calculator. The Chi-square test and I^2^ test were used to evaluate the statistical heterogeneity among the included studies. In the presence of statistical heterogeneity (*p* < 0.05 or I^2^ > 50%) [69], a random effect model was used, otherwise, a fixed-effect model was adopted [70]. For constitution types with sufficient data and adequate quality, pooled effect sizes (OR and 95% CI) were reported. Publication bias was assessed using funnel plots if the minimum number of studies was reached (*n* ≥ 10) [71]. The symmetry of the plots was examined to evaluate potential publication bias.

## 3. Results

### 3.1. Study Selection

**Figure 1:**
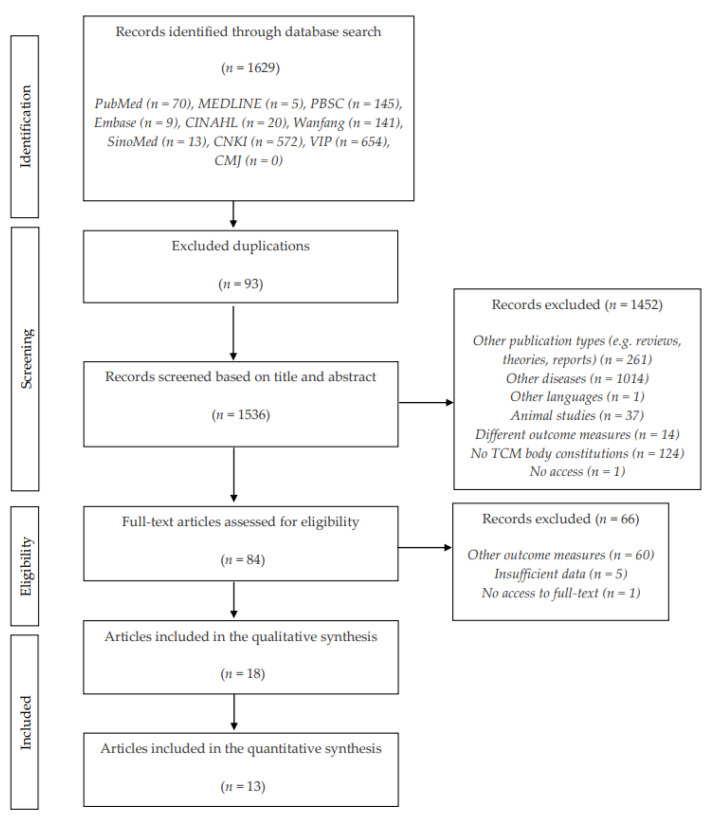
PRISMA flowchart.

A total of 1629 records were retrieved based on the search strategy. After removing duplicated records and reviewing titles and abstracts, 84 potentially relevant articles were identified for further full-text screening. Sixty-six studies were excluded because the authors failed to provide relevant data, or the studies measured different outcomes, and did not meet the inclusion criteria, respectively. Overall, eighteen studies were eligible for inclusion in this systematic review and thirteen studies were eligible for meta-analysis. The study selection process and the rationale for study exclusion is reported in Figure 1.

### 3.2. Characteristics of Included Studies

Details of the included studies are summarized in Table 2. Eleven out of eighteen studies were conducted among diseased populations, which included hospital outpatients and inpatients. Whereas another seven were focused on healthy populations, which included samples from biobank, community or institutional groups. The included studies were published in the past decade, between 2010 and 2021. Five were case-control studies and thirteen were cross-sectional studies. The total sample size was 14,799, with an average sample size of 822. The age of the subjects ranged between eighteen and seventy-five years old. Two studies were focused on female subjects only and the rest were focused on both males and females. All of the studies were conducted in China (*n* = 18). Sixteen studies were written in Chinese and the other two were written in English. The method for depression and TCMBC measurement was based on validated self-reported questionnaires. Four instruments were used to identify depression: Hamilton Depression Rating Scale (HAMD) (Cronbach’s alpha = 0.8 [72]) contributing the most studies (*n* = 6), followed by the Self-Rating Depression Scale (SDS) (Cronbach’s alpha = 0.73) [73] (*n* = 5), Beck Depression Inventory II (BDI-II) (Cronbach’s alpha 0.946 [74]) (*n* = 5) and Chinese Classification of Mental Disorders (CCMD-3) (*n* = 1). For studies using identical depression scales, there were variations for the cut-off points used among the studies. For example, the cut-off points for SDS ranged between 50 and 52. Two instruments used to identify TCMBC included the Chinese Medicine Constitution Questionnaire (CMCQ) (n = 17) (Cronbach’s alphas in each subscale = 0.72~0.80 [75,76]) and the Body Constitution Questionnaire (BCQ) (*n* = 1) (Cronbach’s alpha = 0.8 [55]). Both instruments consist of different subscales to categorize each type of constitution. Participants were categorized according to their highest score among the subscales. Most of the studies (*n* = 16) focused on all nine types of TCMBC while the rest (*n* = 2) focused on specific constitution types.

### 3.3. Quality Appraisal

The quality assessment of the included studies is reported in Table 3. Seven studies were rated as good quality, seven were medium quality and four were poor quality. The NOS scores of all included studies ranged between 5 and 8. The average score for case control studies was 5.2 and for cross-sectional studies was 6.5. Poor quality studies were excluded from the meta-analyses. The inter-rater reliability for this review was 94%.

### 3.4. Systematic Review of Associations between TCMBC and Depression

All nine types of TCMBC were showed to be associated with depression, with QSC contributing the most (*n* = 15), followed by QDC (*n* = 14), BC (*n* = 6), YADC (*n* = 5), YIDC (*n* = 5), BSC (*n* = 3), PDC (*n* = 2), DHC (*n* = 1), and ISC (*n* = 1).

#### 3.4.1. Qi-Stagnation Constitution

Among the studies that revealed a link between QSC and depression, diseased populations were more frequently observed, with a higher prevalence among post-stroke patients [80,81,82,84], followed by cervical cancer patients [78], diabetic patients [86], epileptic patients [87], heart disease patients [62], irritable bowel syndrome (IBS) patients [85] and neurological patients [77]. In addition, the other five studies were carried out among general populations, which included university students [38,60,61,83], and young adults [58]. These studies showed consistent results where QSC significantly predicted depression. Four studies were excluded from the meta-analysis due to poor quality [78,80,82,84] and the other one was excluded due to differences in the study design [81].

#### 3.4.2. Qi-Deficiency Constitution

Among the studies that found a correlation between QDC and depression, eight were conducted on diseased populations, including cervical cancer patients [78], chronic hemodialysis patients [79], diabetic patients [86], epileptic patients [87], heart disease patients [62], IBS patients [85] and post-stroke patients [81,84], Whereas in general populations, most studies focused on university students [38,60,61,83], followed by railway crews [59] and young adults [58]. All studies suggest that QDC is a significant risk factor for depression. Three studies were excluded from the meta-analysis due to poor quality [78,84] and differences in the study design [81].

#### 3.4.3. Yang-Deficiency Constitution

Of the five studies indicating a link between YADC and depression, two were from diseased populations, which included neurological patients [77] and post-stroke patients [84]. The rest were from university students [61,83] and women [35]. The consistent findings suggest that YADC is a risk factor for depression. One study was excluded from the meta-analysis due to poor quality [84].

#### 3.4.4. Yin-Deficiency Constitution

Five studies showing that YIDC is significantly related to depression were conducted among diabetic patients [86], post-stroke patients [82], women [35] and university students [61,83]. There were inconsistent findings among the studies, in which Pang et al. showed that YIDC was negatively associated with depression. While the rest showed that YIDC was positively associated with depression [35,61,83,86]. One study was excluded from the meta-analysis due to poor quality [82].

#### 3.4.5. Blood-Stasis Constitution

Three out of eighteen included studies indicated that BSC was correlated with depression among cervical cancer patients [78], post-stroke patients [80], and university students [83]. Inconsistent findings were observed among the included studies. Two studies found that BSC was negatively correlated to depression [80,83]. In contrast, Ke et al. [78] found that BSC was positively correlated with depression. No meta-analysis was performed for this constitution type because there was only a single study of sufficient quality [83].

#### 3.4.6. Phlegm-Dampness Constitution

Only two studies demonstrated a link between PDC and depression. One was conducted among post-stroke patients [82] and the other among university students [61]. Both studies showed consistent results, suggesting that PDC was a risk factor for depression. No meta-analysis was performed for this constitution type because there was only a single study of sufficient quality [61].

#### 3.4.7. Damp-Heat Constitution

There was one study conducted among young adults which revealed that DHC was correlated with depression [58]. The findings showed that DHC was a risk factor for depression. No meta-analysis was performed for this constitution type due to the insufficient number of studies.

#### 3.4.8. Inherited Special Constitution

Only one study focused on university students found that ISC was related to depression [61]. Their findings suggested that ISC was a risk factor for depression. No meta-analysis was performed for this constitution type due to the insufficient number of studies.

#### 3.4.9. Balanced Constitution

Six out of eighteen included studies demonstrated a relationship between balanced constitution and depression. The majority of the studies were focused on general populations, such as university students [38,60,83] and young adults [58]. The rest were focused on epileptic patients [87] and post-stroke patients [80]. All studies showed consistent results, in which BC was found to be a protective factor for depression. One study was excluded from meta-analysis because of differences in the study design [80].

### 3.5. Meta-Analyses of Association between TCM Body Constitution and Depression

For the association between each type of TCMBC and depression, a meta-analysis was conducted only when there was sufficient data (*n* ≥ 2 and with adequate quality).

#### 3.5.1. Qi-Stagnation Constitution

**Figure 2:**
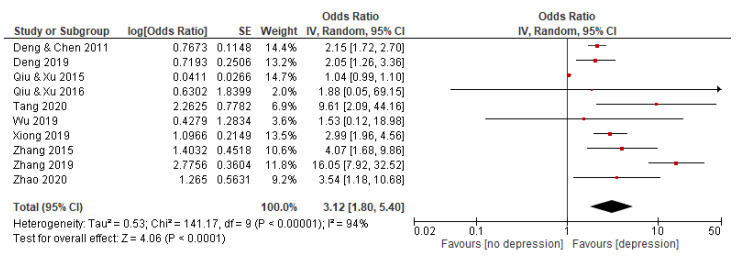
Forest plot of studies on association between QSC and depression. Note: red square represents the result of each study; line represents the 95% CI of the results; diamond represents the pooled results.

Among eighteen included studies, ten studies involving 11,437 subjects reported an association between QSC and depression. The random effects model was used because the statistical heterogeneity of the included studies was significant (I^2^ = 94%). The results showed that the association between QSC and depression was significant, with a pooled OR and 95% CI of 3.12 [1.80–5.40] (see Figure 2).

#### 3.5.2. Qi-Deficiency Constitution

**Figure 3:**
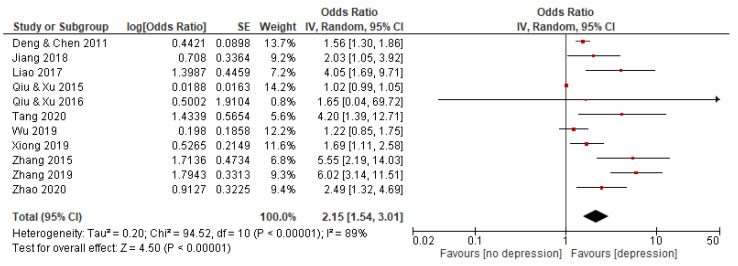
Forest plot of studies on association between QDC and depression. Note: red square represents the result of each study; line represents the 95% CI of the results; diamond represents the pooled results.

Eleven studies with a total of 12,053 subjects reported an association between QDC and depression. The random effects model was used because the statistical heterogeneity of the included studies was significant (I^2^ = 89%). The results showed that the association between QDC and depression was significant, with a pooled OR and 95% CI of 2.15 [1.54–3.01] (see Figure 3).

#### 3.5.3. Yang-Deficiency Constitution

**Figure 4:**
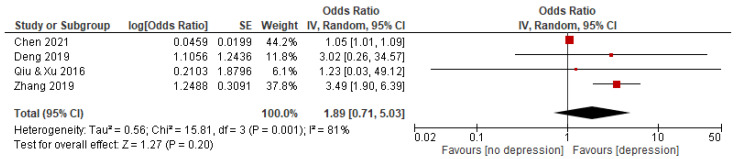
Forest plot of studies on association between YADC and depression. Note: red square represents the result of each study; line represents the 95% CI of the results; diamond represents the pooled results.

Four studies with a total of 2910 subjects reported an association between YADC and depression. The random effects model was used because the statistical heterogeneity of the included studies was significant (I^2^ = 81%). The results showed that the association between YADC and depression was significant, with a pooled OR and 95% CI of 1.89 [0.71–5.03] (see Figure 4).

#### 3.5.4. Yin-Deficiency Constitution

**Figure 5:**
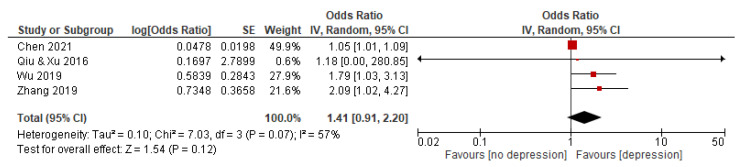
Forest plot of studies on association between YIDC and depression. Note: red square represents the result of each study; line represents the 95% CI of the results; diamond represents the pooled results.

Four studies with a total of 2992 subjects reported an association between YIDC and depression. The random effects model was used because the statistical heterogeneity of the included studies was significant (I^2^ = 57%). The results showed that the association between YIDC and depression was significant, with a pooled OR and 95% CI of 1.41 [0.91–2.20] (see Figure 5).

#### 3.5.5. Balanced Constitution

**Figure 6:**
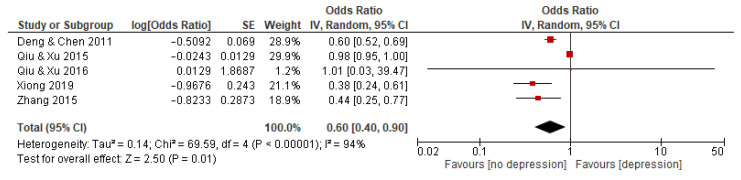
Forest plot of studies on association between BC and depression. Note: red square represents the result of each study; line represents the 95% CI of the results; diamond represents the pooled results.

Five studies with a total of 10,113 subjects reported an association between BC and depression. The random effects model was used because the statistical heterogeneity of the included studies was significant (I^2^ = 94%). The results showed that the association between BC and depression was significant, with a pooled OR and 95% CI of 0.60 [0.40–0.90] (see Figure 6).

### 3.6. Risk of Bias

**Figure 7:**
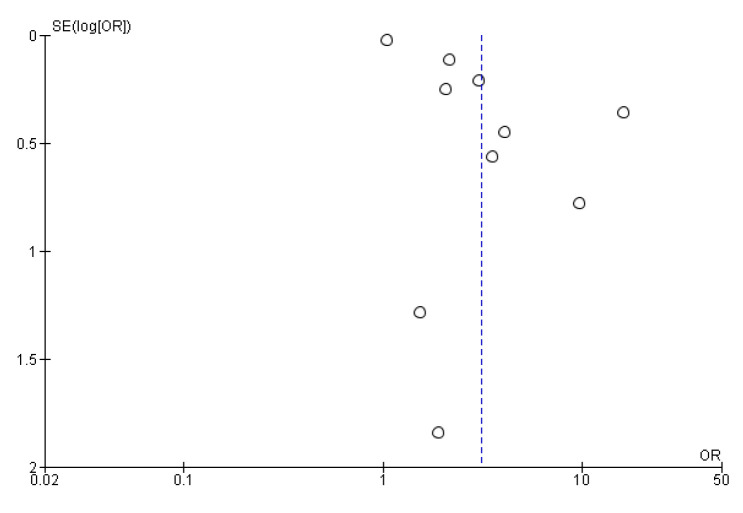
Funnel plot of studies on association between QSC and depression. Note: dot represents individual studies; blue dotted line represents the overall effect.

**Figure 8:**
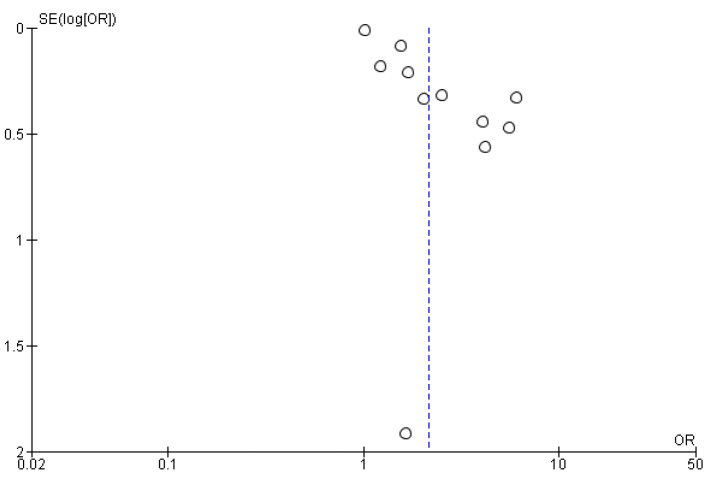
Funnel plot of studies on association between QDC and depression. Note: dot represents individual studies; blue dotted line represents the overall effect.

A visualization of the funnel plots suggests no clear evidence of publication bias (see Figure 7 and Figure 8). For other constitution types, we were unable to perform a publication bias analysis due to the small number of studies [71].

### 3.7. Certainty of Evidence

The detailed GRADE ratings for each meta-analysis are reported in Table 4. As all the included studies in these meta-analyses were observational studies, the level of certainty was initially rated as low. One point was deducted for all meta-analyses due to inconsistency (due to the presence of statistical heterogeneity). However, for the associations of QSC and QDC with depression, the quality of evidence was increased by one point due to the large magnitude of effect. The overall quality of evidence of was rated as very low.

## 4. Discussion

Traditional Chinese medicine body constitutions (TCMBC) are classified based on the harmony and balance of Yin-Yang, Qi and blood within the human body. There are nine types of TCMBC, where Balanced constitution (BC) is a neutral constitution, and the rest are the biased constitutions, namely Qi-stagnation (QSC), Blood-stasis (BSC), Qi-deficiency constitution (QDC), Yin-deficiency constitution (YIDC), Yang-deficiency constitution (YADC), Phlegm-dampness (PDC), Damp-heat constitution (DHC) and Inherited special constitution (ISC) [34]. This review shows that all nine types of TCMBC were associated with depression. Of the nine constitutions, QSC, QDC, BC, YADC, YIDC, BSC and PDC showed significant relationships with depression among both diseased and general populations. QSC, QDC, YADC and YIDC were independent risk factors for depression. When compared to other biased constitutions, people with QSC and QDC were 3.12 and 2.15 times more likely to be depressed, respectively. The strong positive associations between biased TCMBC (e.g., QSC, QDC, YADC and YIDC) and depression could be explained by the interactions between fundamental substances (essence, Qi, blood, body fluids and spirit) and viscera (liver, spleen, lung, heart and kidney). Generally, the blockage of Qi and blood and the abnormal liver functions are considered correlated to depression in TCM.

According to TCM, Qi plays an essential role in propelling, warming and transforming [31,32]. There are variations in the functions of Qi in different viscera. The propelling effect of Qi is responsible for stimulating and maintaining the normal function of internal organs [88]. For example, heart-Qi is responsible for promoting blood circulation [89], whereas liver-Qi regulates the smooth movement of Qi [43,90]. The impaired propelling function of Qi can cause hypofunction of the viscera and subsequent deficiency problems [91]. Furthermore, Qi with a warming effect is called Yang-Qi. In TCM, Yang-Qi in the heart warms and dredges our blood vessels to promote blood circulation [92], while Yang-Qi in the spleen warms and transforms food and water [93,94], ensuring good digestion and absorption [94]. If the warming effect of Qi is weakened, it will result in restricted circulation of Qi and blood, as well as the devitalization of the visceral functions. Additionally, Qi-transformation is vital in maintaining the balance of fundamental substances within our body [32]. For example, Qi is involved in producing and transforming other fundamental substances, such as blood, essence and body fluid. If the transforming function of Qi is weakened, physiological functions will be affected, resulting in various diseases. Overall, a lack or deficiency of Qi will lead to the development of weak immunity. In TCM, the liver plays important roles in regulating emotions and the maintenance of the movement of Qi and blood [31,43,45]. When the liver functions normally, free flow of Qi and blood is maintained, ensuring the transport of essential nutrients to other viscera, which results in good physical and mental health [45]. If the liver’s function of dispersion and dredging is abnormal, the flow of Qi and blood in the body may be obstructed, which can result in various problems, such as insomnia, melancholy, sentimentality, mood swings and even depression [31,43,48,51]. Disruptions of Qi and blood in viscera will affect a person’s mental activities, and abnormal mental activities can affect the Qi and blood in viscera, as well. For example, excessive emotions like panic, stress and sadness can cause dysfunctions of Qi, blood and viscera, and eventually lead to the development of depression [43].

On the contrary, BC is shown to be a significant protective factor against depression. This result was expected, because BC is defined as a neutral and harmonious constitution type, with a balance of Yin-Yang, Qi and blood in the body. People with this constitution usually have an optimistic personality and good adaptability. High optimism helps reduce the incidence of depression because optimistic people often think positively and are more resistant to stress. Moreover, change induces negative emotions, such as stress, anxiety and even depression. People with good adaptability can handle and adapt to changes quickly; their ability to cope with changed or changing situations can subsequently minimize their risk of depression. Besides, people with BC tend to practice healthier lifestyles compared to people with biased TCMBCs. Heathy lifestyles characterized by balanced diet and sufficient exercise are shown to be beneficial for mental health [95].

The strength of this review is that its findings can contribute to the prevention and treatment of depression through the modification of TCMBC. Since this review identified those with QSC and QDC as populations at high risk for depression, we suggest that screening of TCMBC be added to depression screening protocols. When a person is identified as either QSC or QDC, they should be considered at high risk for depression. Since TCMBC is modifiable, depression can actually be prevented before it even develops in people with QSC and QDC. In order to modify these biased constitutions towards a balanced constitution, moderate amount of exercise, such as yoga and cardio, is highly recommended for people with QSC and QDC, as it can help promote the circulation of Qi and blood in the body [31]. Moreover, sleeping at regular times and getting adequate sleep is vital for nourishing the Qi [31]. As for diet, these people should eat a greater variety of foods that can nourish the blood and help with Qi movement, such as dark leafy greens, bean sprouts, berries and red meats [31].

This review has several limitations. First, the application of TCMBC is still in infancy in countries other than China, and the sources of the included studies were all from China. The majority of the included studies were written in Chinese, even though we searched four English databases and six other Chinese databases. This could be due to the lack of application of TCMBC in countries other than China. Currently, there is only one previous study related to TCMBC and depression among non-Chinese populations, conducted among African students studying in China [96]. However, the study is not included in our review due to different outcome measures. Thus, the findings of this review cannot be generalized to other populations. Secondly, the study design of the included studies were mostly cross-sectional studies, followed by case-control studies. No case-control studies were included in the meta-analysis. Hence, the temporal relationship and the causal link between TCMBC and depression is unknown. Third, all the included studies were observational studies using self-reported questionnaires as the measurement tool; therefore, recall bias may be present. Fourth, there was presence of high heterogeneity. This could be due to the variations in sample sizes, sample populations and measurement tools. Fifth, the validation of TCMBC-related questionnaires are limited to the Chinese and English languages only. This could explain why TCMBC is not widely applicable in other countries. Further research is likely to significantly impact our confidence regarding the estimated effects, as the levels of certainty of the current review measured by GRADE were rated as low and very low, respectively.

## 5. Conclusions

This review demonstrated that Qi-stagnation, Qi-deficiency, Yang-deficiency, Yin-deficiency and Balanced constitutions are significant predictors for depression, of which, Balanced constitution is the protective factor. Because most of the included studies were cross-sectional, we suggest that more case-control and cohort studies be analyzed to confirm the association between TCMBC and depression.

## Figures and Tables

**Table 1:** Search terms.

Concept	Search Terms
**Depression**	depression OR depressive disorder OR *yuzheng*
**TCMBC**	traditional Chinese medicine constitution OR traditional Chinese medicine body constitution

Note: *yuzheng* refers to depression in traditional Chinese medicine.

**Table 2:** Characteristics of included studies.

Author, Year	Study Design	Study Subjects	Sample Size	Age Range	Sex	Study Area	Depression Measurement	Body Constitution Measurement	Constitution (Specific/All Nine)	Main Findings	Effect Sizes OR [95% CI]
**Diseased populations**											
**Deng et al., 2019** [77]	CS	Neurological patients	132	42.51 ± 6.03	U	China	SDS	CMCQ	All	YADC and QSC were independently correlated with depression state.	**YADC**: 3.021 [0.264–5.619]; **QSC**: 2.053 [1.256–3.251]
**Ke et al., 2019** [78]	CC	Cervical cancer patients	289	56.84 ± 14.47	F	China	SDS	CMCQ	All	BSC, QSC and QDC were risk factors of depression in patients with cervical cancer.	**BSC**: 2.923 [1.986–3.864]; **QSC**: 4.158 [1.014–9.869]; **QDC**: 1.875 [1.067–2.024]
**Liao et al., 2017** [79]	CS	Chronic hemodialysis patients.	467	63 ± 12	U	China	BDI-II	CCMQ	All	QDC is associated with depression in chronic HD patients	**QDC**: 4.05 [1.69–9.72]
**Liu & Li, 2010** [80]	CC	Post-stroke depressed patients	90	40–67	U	China	HAMD	CMCQ	QSC, BSC, BC	PSD patients with QSC have a higher depression tendency compared to BSC and BC.	**QSC**: 34.544 [10.325–117.219]; **BCS**: 0.192 [0.056–0.682]; **BC**: 0.234 [0.086–0.632]
**Liu et al., 2019** [81]	CC	Patients with post-cerebral infarction	252	18–75	U	China	HAMD	CMCQ	All	For post-cerebral infarction, QSC and QDC are the major body constitutions which can lead to depression.	**QSC**: 3.865 [2.124–4.385]; **QDC**: 2.127 [1.985–3.654]
**Pang et al., 2018** [82]	CC	Post-first ischemic stroke patient	207	61.94 ± 13.54	U	China	HAMD	CMCQ	All	Post-first ischemic stroke depression is closely related to constitution types of QSC, PDC and YIDC.	**QSC**: 4.58 [1.500–14.001]; **PDC**: 2.98 [1.010–8.605]; **YIDC**: 0.317 [0.122–0.826]
**Sun et al., 2012** [83]	CC	Post stroke patients	353	61.4 ± 9.00	U	China	CCMD-3	CMCQ	All	QSC, YADC and QDC were risk factors for depression among post stroke patients	**QSC**: 2.794 [1.137–7.171]; **YADC**: 3.757 [1.137–12.118]; **QDC**: 3.840 [1.357–12.808]
**Tang et al., 2020** [84]	CS	Irritable bowel syndrome patient	147	≥18	U	China	HAMD	CMCQ	All	There is a certain correlation between TCMBC and depression in IBS patients, in which QDC and QSC are more likely to produce depression.	**QDC**: 4.195 [1.385–12.708]; **QSC**: 9.607 [2.09–44.157]
**Wu et al., 2019** [85]	CS	Diabetic patients	214	52.31 ± 12.25	U	China	HAMD	CMCQ	All	There exists a correlation between depression after T2DM and Chinese medicine constitution to some extent.	**YIDC**: 1.793 [1.027–2.125]; **QSC**: 1.534 [0.124–0.863]; **QDC**: 1.219 [0.847–2.121]
**Zhao et al., 2020** [62]	CS	Patients with coronary heart disease	160	58.41 ± 6.81	U	China	SDS	CMCQ	All	QDC and QSC were independent risk factors for depression in patients with CHD.	**QDC**: 2.491 [1.324–4.731]; **QSC**: 3.543 [1.175–10.638]
**Zhang et al., 2015** [86]	CS	Adult patients with epilepsy	209	18–70	U	China	HAMD	CMCQ	All	QDC and QSC are prone to depression in adult patients with epilepsy, while BC is the protective factor.	**QDC**: 5.549 [2.194–14.039]; **QSC**: 4.068 [1.678–9.861]; **BC**: 0.439 [0.250–0.771]
**Healthy populations**											
**Chen et al., 2021** [35]	CS	Women from Taiwan Biobank	1423	30–70	F	China	NS	BCQ	YADC, YIDC, PDC	Women who have YADC or YIDC were more prone to depression.	**YADC**: 1.047 [1.007–1.089]; **YIDC**: 1.049 [1.009–1.090]
**Deng & Chen, 2011** [58]	CS	General population	7506	≥18	U	China	SDS	CMCQ	ALL	People with QDC, DHC and QSC had high tendency of depression, while people with BC had a lower tendency of depression.	**BC**: 0.601 [0.525–0.689]; **QDC**: 1.556 [1.305–1.855]; **DHC**: 2.140 [1.705–2.686]; **QSC**: 2.154 [1.720–2.697]
**Jiang et al., 2018** [59]	CS	Beijing Railway crews	281	20–35	U	China	SDS	CMCQ	ALL	QDC was significantly correlated to depression among railway crews.	**QDC**: 2.03 [1.05–3.94]
**Qiu & Xu, 2015** [60]	CS	University students	764	NS	U	China	BDI	CMCQ	ALL	BC is the protective factor for depression among the students, while QDC and QSC are the risk factors.	**BC**: 0.976 [0.978–1.001]; **QDC**: 1.019 [0.987–1.015]; **QSC**: 1.042 [0.989–1.016]
**Qiu & Xu, 2016** [87]	CS	University students	684	NS	U	China	BDI	CMCQ	ALL	QDC, YADC, YIDC, QSC, BC, and BSC were the predictors for depression.	**BC**: 1.013 [−0.026, −0.001]; **QDC**: 1.649 [0.039–0.061]; **YADC**: 1.234 [−0.031, −0.011]; **YIDC**: 1.185 [0.005–0.029]; **QSC**: 1.878 [0.051–0.075]; **BSC**: 1.174 [−0.030, −0.003]
**Xiong et al., 2019** [38]	CS	University students	950	NS	U	China	BDI-II	CMCQ	ALL	BC is the protective factor while QDC and QSC are the risk factors for depression among university students.	**BC**: 0.380 [0.236–0.610]; **QDC**: 1.693 [1.111–2.578]; **QSC**: 2.994 [1.965–4.561]
**Zhang et al., 2019** [61]	CS	University students	671	20.40 ± 1.48	U	China	BDI-II	CMCQ	ALL	Results showed that YADC, YIDC, QDC, PDC, ISC and QSC were risk factors of depression.	**YADC**: 3.486 [1.902–6.389]; **YIDC**: 2.085 [1.018–4.267]; **QDC**: 6.015 [ 3.142–11.514]; **PDC**: 2.556 [1.145–5.707]; **ISC**: 8.888 [4.406–17.929]; **QSC**: 16.049 [7.919–32.525]

Note: TCMBC, Traditional Chinese medicine body constitution; CS, cross-sectional study; CC, case-control study; NS, not specify or report in the article; U, unisex; F, female; SDS, Self-Rating Depression Scale; BDI-II, Beck Depression Inventory II; HAMD, Hamilton Depression Rating Scale; CCMD-3, Chinese Classification of Mental Disorders; CMCQ, Chinese Medicine Constitution Questionnaire; BCQ, Body Constitution Questionnaire; QSC, Qi-stagnation constitution; BSC, Blood-stasis constitution; BC, Balanced constitution; YADC, Yang-deficiency constitution; YIDC, Yin-deficiency constitution; PDC, Phlegm-damp constitution; HD, hemodialysis; PSD, Post-stroke depression; IBS, Irritable bowel syndrome; T2DM, Type 2 diabetes mellitus; CHD, coronary heart disease.

**Table 3:** Quality assessment of the included studies by the Newcastle–Ottawa Scale (NOS).

Author, Year	Selection	Comparability	Outcome	Total Score
**Chen et al., 2021** [35]	★★	★★	★★★	7
**Deng & Chen, 2011** [58]	★	★	★★★	5
**Deng et al., 2019** [77]	★	★★	★★★	5
**Jiang et al., 2018** [59]	★★	★	★★★	6
**Ke et al., 2019** [78] **^cc^**	★★★	★	★	5
**Liao et al., 2017** [79]	★★★	★★	★★★	8
**Liu & Li, 2010** [80] **^cc^**	★★★	★	★	5
**Liu et al., 2019** [81] **^cc^**	★★★	★	★★	6
**Pang et al., 2018** [82] **^cc^**	★★★	★	★	5
**Qiu & Xu, 2015** [60]	★★	★	★★★	6
**Qiu & Xu, 2016** [87]	★	★	★★★	6
**Sun et al., 2012** [83] **^cc^**	★★★	★	★	5
**Tang et al., 2020** [84]	★★	★	★★★	6
**Wu et al., 2019** [85]	★★	★	★★★	7
**Xiong et al., 2019** [38]	★★	★	★★★	7
**Zhang et al., 2015** [86]	★★	★	★★★	6
**Zhang et al., 2019** [61]	★★	★	★★★	7
**Zhao et al., 2020** [62]	★★★	★	★★★	7

Note: ^cc^, case control study. A maximum of one star can be awarded for each numbered item within the selection and exposure section. A maximum of two stars can be given for the comparability section. The maximum scores for case-control studies and cross-sectional studies are 9 and 10, respectively. For the quality of case-control studies, it was considered as poor if the score was 0 to 5 and good if the score was 6 to 9. For the quality of cross-sectional studies, it was considered as poor if the score was 0 to 4, medium if the score was 5 to 6, good if the score was 7 to 8, and very good if the score was 9 to 10.

**Table 4:** GRADE assessment of all meta-analyses.

Number of Studies	Study Design	Risk of Bias	Inconsistency	Imprecision	Indirectness	Publication Bias	Other Considerations	Number of Subjects	Effect Sizes [95% CI]	Overall Quality of Evidence
**QSC**										
**10**	Observational studies	Not serious	Serious ^a^	Not serious	Not serious	Not detected	Very strong association ^c^	11,437	3.12 [1.80, 5.40]	Low
**QDC**										
**11**	Observational studies	Not serious	Serious ^a^	Not serious	Not serious	Not detected	Very strong association ^c^	12,053	2.15 [1.54, 3.01]	Low
**YADC**										
**4**	Observational studies	Not serious	Serious ^a^	Not serious	Not serious	Not detected ^b^	None	2910	1.89 [0.71, 5.03]	Very low
**YIDC**										
**4**	Observational studies	Not serious	Serious ^a^	Not serious	Not serious	Not detected ^b^	None	2992	1.41 [0.91, 2.20]	Very low
**BC**										
**5**	Observational studies	Not serious	Serious ^a^	Not serious	Not serious	Not detected ^b^	None	10,113	0.60 [0.40, 0.90]	Very low

Note: ^a^ significant heterogeneity was detected (I^2^ > 50%); ^b^ unable to perform publication bias assessment due to small number of studies; ^c^ magnitude of the effect was large (OR > 2); Low quality indicated that the authors’ confidence in the effect estimate is limited; Very low quality indicated that the authors have very little confidence in the estimated effect.

## Data Availability

Not applicable.
